# Dynamics of T-Junction Solution Switching Aimed at Patch Clamp Experiments

**DOI:** 10.1371/journal.pone.0133187

**Published:** 2015-07-15

**Authors:** Jerónimo A. Auzmendi, Mariano Smoler, Luciano Moffatt

**Affiliations:** Instituto de Química Física de los Materiales, Medio Ambiente y Energía. Facultad de Ciencias Exactas y Naturales, Universidad de Buenos Aires, Buenos Aires, Argentina; University of Antwerp, BELGIUM

## Abstract

Solutions exchange systems are responsible for the timing of drug application on patch clamp experiments. There are two basic strategies for generating a solution exchange. When slow exchanges are bearable, it is easier to perform the exchange inside the tubing system upstream of the exit port. On the other hand, fast, reproducible, exchanges are usually performed downstream of the exit port. As both strategies are combinable, increasing the performance of upstream exchanges is desirable. We designed a simple method for manufacturing T-junctions (300 μm I.D.) and we measured the time profile of exchange of two saline solutions using a patch pipette with an open tip. Three factors were found to determine the timing of the solution switching: pressure, travelled distance and off-center distance. A linear relationship between the time delay and the travelled distance was found for each tested pressure, showing its dependence to the fluid velocity, which increased with pressure. The exchange time was found to increase quadratically with the delay, although a sizeable variability remains unexplained by this relationship. The delay and exchange times increased as the recording pipette moved away from the center of the stream. Those increases became dramatic as the pipette was moved close to the stream borders. Mass transport along the travelled distance between the slow fluid at the border and the fast fluid at the center seems to contribute to the time course of the solution exchange. This effect would be present in all tubing based devices. Present results might be of fundamental importance for the adequate design of serial compound exchangers which would be instrumental in the discovery of drugs that modulate the action of the physiological agonists of ion channels with the purpose of fine tuning their physiology.

## Introduction

Ion channels are membrane proteins that regulate the passage of ions through the cellular membrane according to different stimuli, such as the binding of a protein-specific ligand, the membrane potential, the membrane tension and the temperature [1, 2]. One important problem in ion channel biophysics consists in understanding the molecular mechanisms that couple the stimulus sensor to the gate of the channel pore [3–7]. The patch clamp technique is employed to measure the current generated by one or many channels in a controlled preparation [8, 9]. Recent efforts lead to a much better control in the presentation of the ligand. In our previous work we applied ligand pulses of 0.2 ms, separating binding from gating on purinergic receptors and we revealed the existence of an intermediate state between them [10]. Encouraging results of our group [11] suggest that it would be possible to obtain pulses ten times shorter necessary to resolve this state in nicotinic or glutamatergic receptors in the near future [12, 13]. However, in order to understand the role of drugs in channel activity, the application of a single compound at a time is not enough. It would be necessary to apply multiple compounds on the same channel preparation in fast succession [14].

From a drug discovery point of view, ion channels are important targets: they regulate a large number of physiological processes and they are involved in many pathologies [15–17]. Ion channels had been much more difficult to screen than soluble proteins; the gold standard assay for assessing their activity, the patch clamp technique, requires a highly skilled operator. Thanks to the invention of the planar patch clamp, automated patch clamp systems have become available increasing the number of targets against which one drug can be tested [17–19]. In these commercial systems, the glass recording pipette has been replaced by a planar surface. In some systems, such as SyncroPatch96 or Patchliner, compounds are applied with the aid of fluid handling robots [20–22], while other parallel systems such as IonFlux use a microfluidic system [23–25]. The functioning principle of these high-throughput systems is based on the parallel application of compounds on multiple samples. Another way to increase the data acquisition rate consists in the successive application of compounds to each sample [26, 27].

Drug application is implemented by driving the patch preparation across the interface between the exchanged solutions. A distinction of solution exchangers can be made according to whether the interface is formed upstream or downstream to the exit port of the perfusion tube. In upstream exchangers a transient interface, transversal to the flow, is formed at a junction inside the tubing system [14, 28, 29]. In downstream exchangers, a stationary interface, parallel to the flow direction is formed right at exit port [10, 26, 27]. Upstream exchangers are operated with the aid of a solenoid or a pinch valve that alternates the flow from one line to the other. Downstream exchangers either deflect the interface by altering the flow of one of the streams, or move the interface by transversally moving the application device with a piezo or stepper motor [10, 11]. Ultra-Fast solution exchanges can be obtained by using downstream exchangers [11]. By setting a large number of parallel laminar flows and shifting the sample across them it is possible to rapidly switch between 32 solutions [26, 27]. Much slower exchanges, in the seconds range, can be achieved by alternating between two or more solutions with the aid of an upstream tee or manifold junction. However, if the tube junction is placed close to the exit port, faster exchanges are feasible. This is the case of the Multi-barrels Perfusion Pencil [30], which offers upstream exchanges in the millisecond range.

Here we present what we think is the first attempt to characterize the behavior of upstream transversal interfaces. We designed a simple method for manufacturing 300 μm internal diameter T-junctions and we measured the time profile of upstream exchange of two saline solutions using a patch pipette with an open measuring tip as the solution concentration sensor. We found that pressure, travelled distance and off-center distance were fundamental parameters to determine the timing of the solution switching.

## Materials & Methods

### Low volume T-junctions manufacture

**Fig 1:**
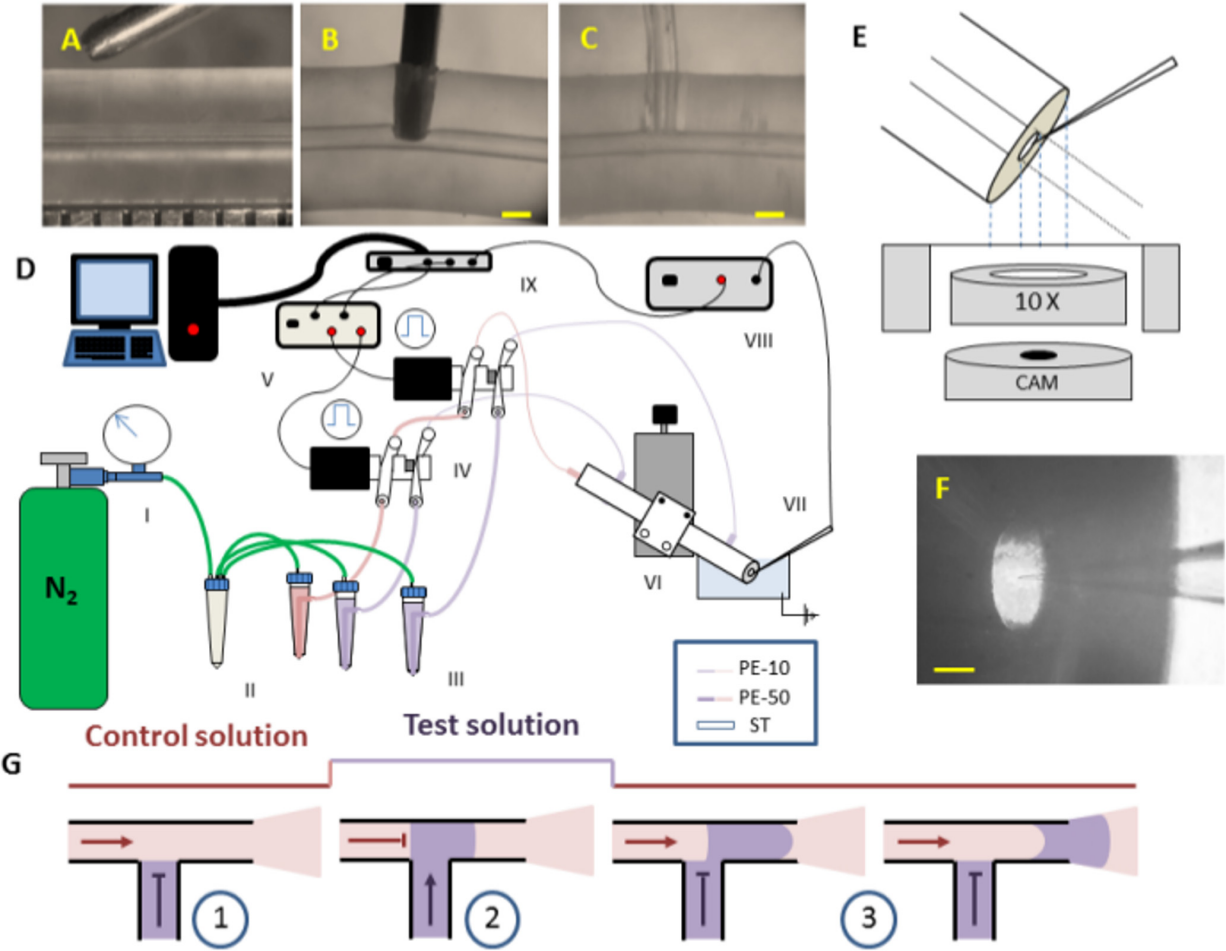
T-junction manufacture and operation. (A) Silicone tubing and punch. A 0.5 mm interval ruler is apparent on the bottom. (B) A hole is drilled perpendicular to the central hole. (C) After inserting a polyethylene tubing the T-junction is completed. (D) General device. *I*. N_2_ tank. *II*. Filter *III*. Pressurized reservoir of solution. *IV*. 3-way solenoid pinch valves. *V*. A 24 V custom made valve driver. *VI*. Merger system of two T-junctions staked in XYZ translator. *VII*. Open tip recording pipette. *VIII*. Patch clamp amplifier. *IX*. A multifunction data acquisition connected to PC. (E) Schematic representation of experimental configuration. (F) Image of experimental configuration. The image was taken using a web camera coupled to the setup microscope. (G) Operation of the solution switching. A pulse of test solution (violet) was inserted into the control solution (pink).

T-junctions were built on 0.30 mm ID (Altec Products LtdBude, United Kingdom) silicone tubing (ST). A punch (Fig 1A) was made out of a 23 G syringe by cutting and filing the tip and grinding the blade with an Arkansas stone. The ST was drilled with the punch perpendicular to the central hole (Fig 1B); to prevent the collapse of the drilled holes, pressurized N_2_ was injected from one end of the tubing while the other end was sealed. Silicone residues were removed with forceps. After all perforations were made, a piece of 0.28 mm ID polyethylene tubing (PE-10 Intramedic, Becton Dickinson) was placed in each one of the punctured holes using a fine-tipped forceps to form T- connectors (Fig 1C). All the T-junctions were secured with epoxy glue (Poxipol, Acapol, Buenos Aires, Argentina).

### Experimental device & data acquisition

The experimental configuration was set to characterize the dynamics of solution switching. It consisted of a solution delivery system, a recording system and an interface to the computer (Fig 1D). In all the experiments we switched between a control and a test solution. A patch pipette connected to a patch clamp setup was used as a real time sensor of the electrolyte concentration. Solution switch was carried out by operating a 3-way pinch valve. The low volume, custom manufactured, T-junction merged both flows paths. The T-junction was positioned at different distances from the application tip. The patch pipette was placed at 0.1 mm of the application tip.

As pictured in Fig 1D, the solution delivery system consisted of *i*) a supplying system that provided pressurized solutions *ii*) a selection system that operated the solutions exchanges *iii*) a merger system that collected the different solutions input in a single output. The supplying system included a solution reservoir (15 ml conic tubes) for each solution which was directly connected via pneumatic air tubing to a N_2_ tube pressurized at 0.1, 0.2 or 0.4 bar. We did not use regulator valves to avoid ripple fluctuations in the flow. No drop in the pressure of the tube was detected in the course of each experiment. In this way we obtained stable solution flows with velocities that could be set between 10 and 50 cm/s.

The selection system included a 3-way solenoid pinch valve (coil Z036S, Sirai Elettromeccanica, Bussero, MI, Italy) that simultaneously closed one flow path and opened the other. Activation of the solenoid moved a ferromagnetic plunger that compressed one of two 40 mm pieces of ST while releasing the other. A 24 V custom made valve driver (Fig 1D, *V*) triggered the valve upon the control of a computer. Test solutions flowed from reservoirs to the silicone tubing system through polyethylene tubing of ID 0.58 mm (PE-50, Warner Instruments, Hamden, CT). PE-50 tubing was connected to silicone tubing via a 20 mm piece of PE-10.

We tested a merger system of two T-junctions (Fig 1D, *VI)* attached to a single 90 mm long ST. The T-junctions were positioned at 20 and 70 mm from the extreme of the tubing that worked as the application tip. The other extreme was connected via PE-10 tubing to the control solution. PE-10 tubing was used to connect both T-junctions to the test solution. The length of the three PE-10 tubing was adjusted so the distance between the solenoid valve and the application tip was always 200 mm.

We recorded the spatial and temporal profiles of the solution exchanges at 0.1, 0.2 and 0.4 bar while varying the distances between the T-junctions and the application tip. Initially, the T-junctions were positioned at 20 and 70 mm from the application tip. Subsequently, the application tip was cut with a cutter so to reduce its distance from the T-junctions to 1 and 51 mm, respectively, while the PE-10 tubing was extended accordingly. We repeated for 1 and 51 mm the same series of experiment that we previously did with 20 and 70 mm. The exchanger system was mounted in a holder attached to a 3D translation stage (461-XYZ-M, Newport, Irvine, CA). The angle between the exchanger system and the recording pipette was set at 105° (Fig 1E).

The recording system comprised a patch pipette (Fig 1D, VII) on a headstage which was mounted on a motorized 3 dimension manipulator (860, Newport) and connected to a patch clamp amplifier (2400, A-M Systems, Sequim, WA; see Fig 1D, *VIII*). All experiments were performed using recording pipettes (GC150F-7.5, Harvard Apparatus, Holliston, MA) pulled in a micropipette horizontal puller (P-1000, Sutter Instrument, Novato, CA) to a resistance of 10–15 MΩ and filled with an internal solution containing (in mM) 145 CsF, 5 NaCl, 1.3 MgCl, 10 HEPES. Control and test solutions were 150 and 15 mM NaCl respectively in HEPES buffer 10 mM. The pH of all solutions was adjusted to 7.4 with aqueous HCl. Experimental data was recorded to 10 KHz, filtered to 1 KHz and voltage holding was -100 mV. A multifunction data acquisition (NI USB 6259, National Instruments, Austin, TX) was used for data acquisition and valve operation (Fig *1*D, *IX*).

### Positioning of the recording pipette

The application tip was set at 40 degrees from the measuring table and positioned with the aid of a xyz-translator stage at the center of the field of the microscope. The recording pipette was set at 105 degrees form the application tip and initially positioned at the top center of the stream. From there we looked for the optimal point and the position of the shortest exchange time by moving with 5 μm steps the recording pipette and testing the exchange time at each point. First we looked in the vertical axis always at the same geometrical distance from the application tip; once we found the optimal vertical point we adjusted the position at the lateral axis. The exchange time was determined from 10 to 90% of the maximal response. Measurements were done at the optimal point or away from the optimal point by displacing the recording pipette at the lateral axis.

## Results

Patch clamp kinetic studies rely on some perfusion device to apply different drugs on the patch membrane. In a similar way we changed the sodium chloride concentration that reaches the open measuring tip of a pipette. In this way we characterized the solution switcher response by measuring the changes in the conductivity of the solution. Two solutions met at the T-junction: one was continuously flowing, the other was static (Fig 1G1). After solution switching occurred, the interface between the formerly and currently flowing solutions started moving towards the application tip (Fig 1G2). No change in concentration occurred at the measuring tip before the interface traveled the distance between the T-junction and the application tip (Fig 1G3).

### Response at the optimal point

**Fig 2:**
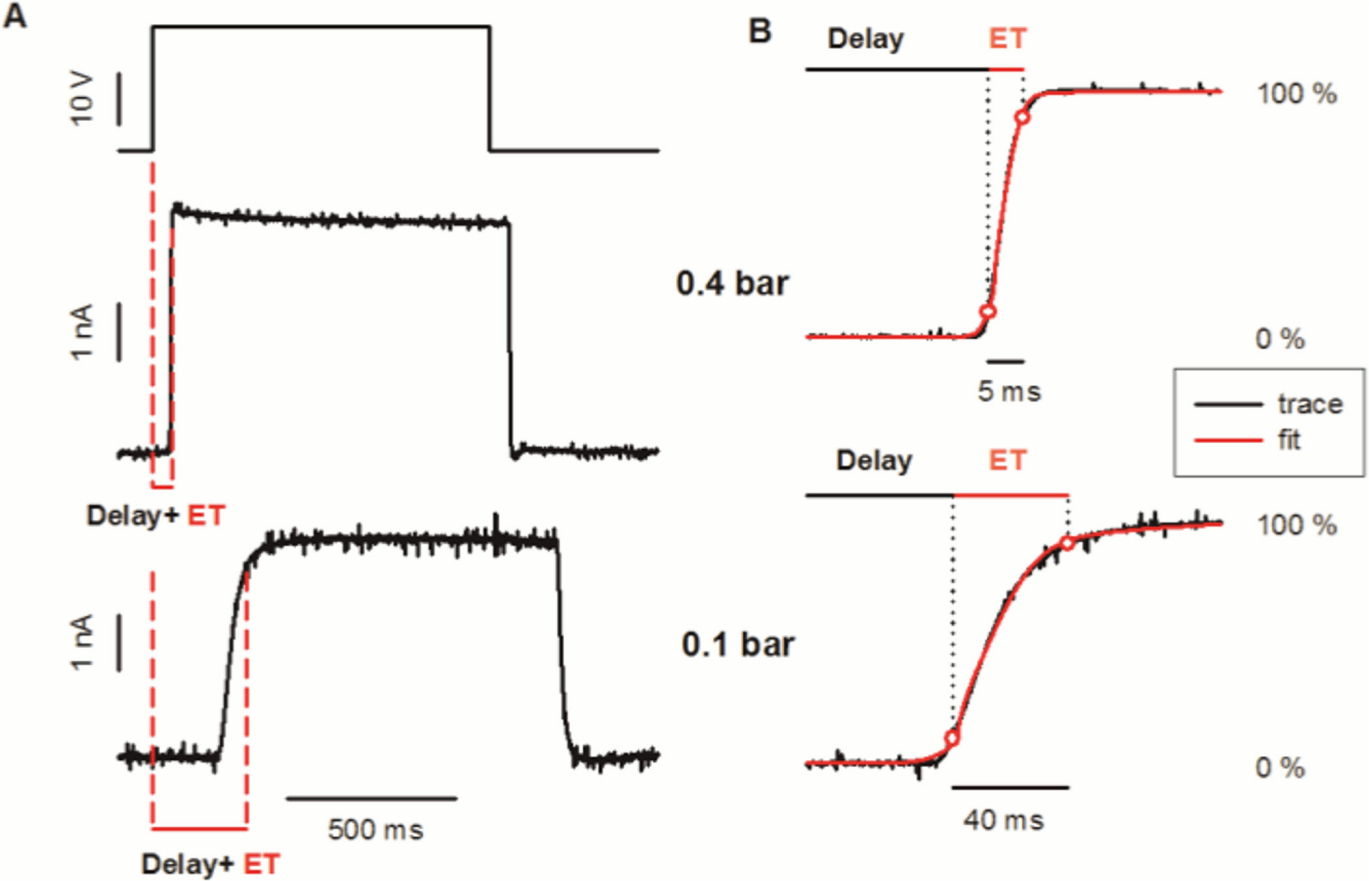
Time course of the solution exchange at two propelling pressures for a constant travelled distance of 20 mm at the optimal position. (A) Complete exchange. From top to bottom: pinch valve voltage command; measured response at 0.4 bar; idem at 0.1 bar. The current was measured while holding the pipette at -100 mV. Dashed line indicates t_on_ and t_90_. (B) Detail of the forward exchange. Measured current (black) and fitted equation (red) at 0.4 bar; idem at 0.1 bar; fitted equation. Red circles indicate the t_10_ and t_90_. Delay time and exchange time (ET) are defined as t_10_-t_on_ and t_90_-t_10_, respectively.

We first studied how different factors affected the solution exchange dynamics after placing the recording pipette in the central point of the stream at 100 μm of the application tip. Fig 2 shows the exchange in a single T-junction at two different propelled pressures. Pulses without oscillations or artifacts were obtained in both cases (Fig 2A). An increase in the pressure produced a decrease in both the delay (t_10_-t_on_) and the exchange time (ET t_90_-t_10_). The complex temporal profile of the solution exchange was successfully approximated by an empirical equation that results from the weighed sum of the error function (erf) and the exponential (exp) of the square root of time.

$$y=\alpha \left[\frac{1}{2}+\frac{1}{2}\text{erf}\left(\frac{t-t_{1/2}}{\sigma }\right)\right]+\left(1-\alpha \right)\left[1-\text{exp}\left(\sqrt{\frac{\text{max}\left(t-t_{0},0\right)}{\tau }}\right)\right]$$

where α indicates the relative weight of the error function, t_1/2_ and t_0_ allow to adjust the delay and sigma and tau account for the rise time. The experimental data was fitted with this equation at different propelled pressures (R^2^ = 0.981 at 0.1 bar & R^2^ = 0.987 at 0.4 bar) (Fig 2B). The error function component increased with pressure (α_0.1_ = 0.618, α_0.4_ = 0.814).

**Fig 3:**
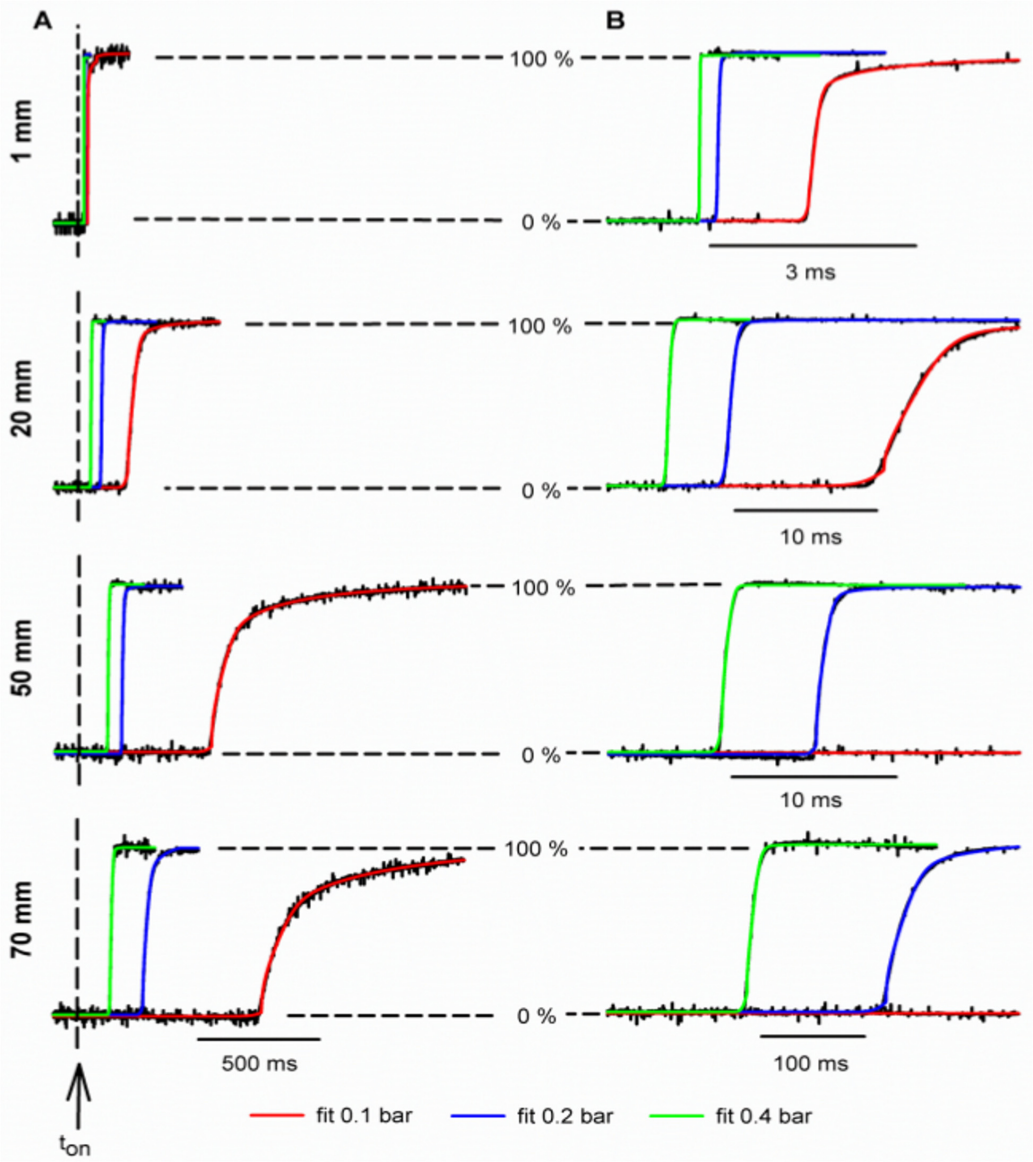
Time course of the forward exchange at different distances (horizontal panels) and pressures (line color) at the optimal position. Experimental data is indicated on black; fits to the equation (same as in Fig 2) are indicated on color. (A) The delay between the time of valve activation (arrow, t_on_) and the exchange increased with both distance and pressure. (B) Expanded time scales showed that the fit covered the data in all cases. The correlation coefficients for 1 mm were the following: 0.986, 0.980 and 0.974 for 0.1, 0.2 and 0.4 bar respectively. For 20 mm, they were: 0.984; 0.988 and 0.987 while for 51 mm: 0.985, 0.975 and 0.982. Finally for 70 mm were: 0.983, 0.982 and 0.980.

To characterize the changes that occur at this transient interface along its way we placed the T-junction at 1, 20, 51, 70 mm of the tip and measured the exchange dynamics (Fig 3). We also studied the effect of the velocity of the fluid on the interface by applying different propellant pressures (Fig 3, trace color indicates pressure). A delay between the time of valve activation and the time when changes in concentration were detected at the measuring tip was always clear; this delay increased both with increasing distance (Fig 3A, compare different panels) and with decreasing propellant pressure (Fig 3A, compare different plots at each panel). The exchange time increased in a similar way (Fig 3B). The colored fit lines covered the black data traces in Fig 3; the correlation coefficient indicated a good fit (R^2^ > 0.97) in all cases. For the same pressure, an increase in the distance traveled decreased α, the fraction of the response that is described by an error function, at the same propelled pressure (Table 1). Furthermore, the increased flow rate increased α (Table 1).

**Table 1:** Fraction of response that is described by an error function (α).

Pressure	Distance (mm)			
(bar)	1	20	51	70
0.1	0.694 ± 0.071	0.605 ± 0.013*	0.301 ± 0.015***	0.273 ± 0.273***
0.2	0.779 ± 0.043	0.817 ± 0.018	0.669 ± 0.015***	0.481 ± 0.481***
0.4	0.861 ± 0.037	0.794 ± 0.015	0.732 ± 0.018***	0.635 ± 0.635***

Table represents the variation of α with both pressure and distance traveled for interface. Data of α was listed as mean ± SD (n = 4) and were statistically analyzed by two-way ANOVA. The interaction between two factors were significant (P < 0.0001) and F = 25.07. The comparisons were made using Bonferroni post-test against the 1 mm column values with a significance level of P < 0.05 (*) and P < 0.001 (***).

**Fig 4:**
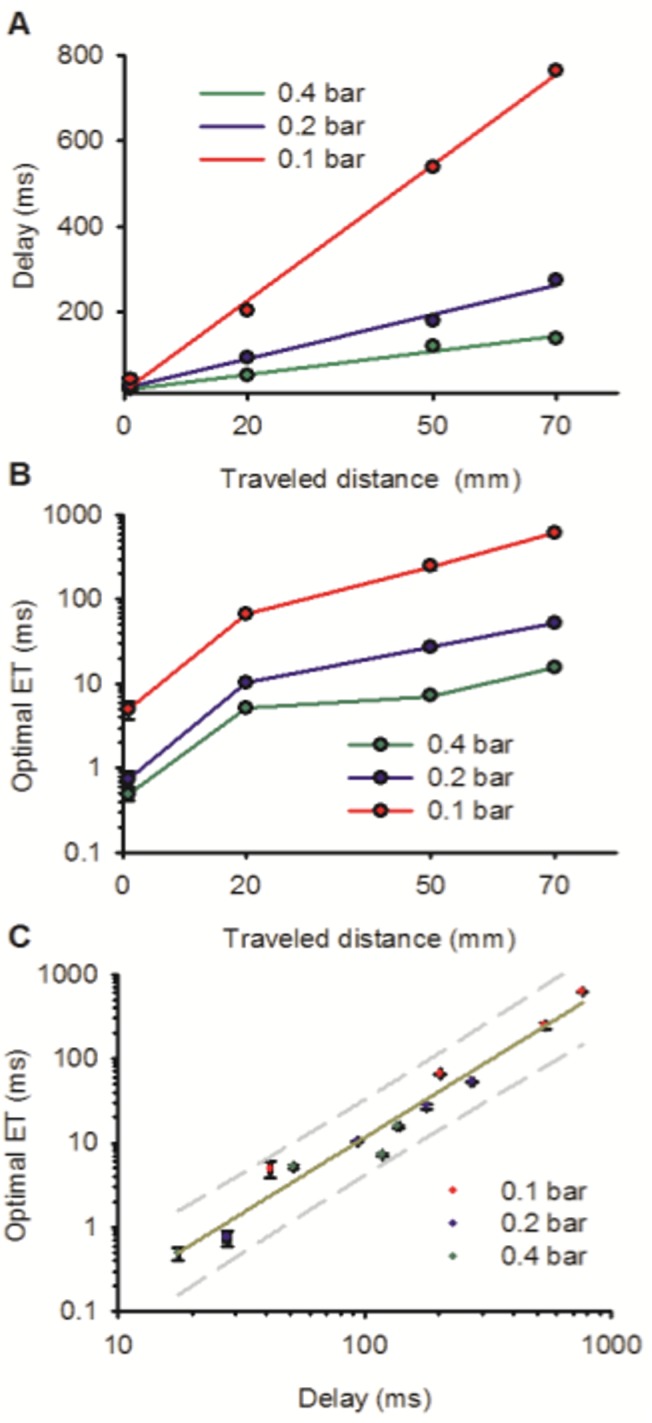
Analysis of the data at the optimal point. (A) Linear plot showing that the delay time increased linearly with the traveled distance at each propelled pressure. (B) Semi-log plot showing the relationship between the exchange time (t_10-90_) and the distance for each propelled pressure. (C) Log-log plot showing a power relationship that describes the exchange time as a function of the delay time for all distances and propelled pressures. Dashed line indicates the 95% prediction interval of a linear regression on the logarithms.

Fig 4*A* shows how the delay in the response increased linearly with distance. At 0.1 bar it increased from 41.44 ± 0.67 ms (n = 4) at 1 mm to 763.32 ± 0.91 ms (n = 4) at 70 mm. At 0.4 bar it increased from 17.50 ± 0.10 ms (n = 5) to 136.39 ± 1.59 ms (n = 4). A linear fit of the delay with distance allowed us to calculate the velocity of the fluid for each N_2_ pressure: 0.492 ± 0.003 m/s at 0.4 bar; 0.267 ± 0.001 m/s at 0.2 bar and 0.098 ± 0.002 m/s at 0.1 bar. This increase in velocity with the pressure was approximately linear (R^2^ = 0.98). Calculated Reynolds numbers ranged from 30 to 150 which are indicative of a laminar flow regime.

Fig 4B shows how the exchange time increased either with increasing distance or with decreasing pressure. Exchange times at 0.4 bar increased from 0.48 ± 0.08 ms (n = 4) at 1 mm to 15.59 ± 0.60 ms (n = 5) at 70 mm; whereas at 0.1 bar they increased from 4.91 ± 1.16 ms (n = 4) to 618.18 ± 7.88 ms (n = 4). A simple diffusion of the interface cannot explain this huge increase; some convective mixing mechanism is clearly involved. The exchange profile becomes more complex with the distance and decreased pressure (Fig 3).

A log-log plot of the exchange rate vs. the delay (Fig 4C) shows that all pressures and distances fall roughly on the same line. A power relationship with a coefficient of 1.81 ± 0.12 described 96% of the variation in the logarithm of exchange time. That is, if we double the time of the solution travel, we expect to approximately quadruple the time for the solution to exchange, no matter if achieved that by doubling the distance or by halving the flow velocity. The delay time appear as the main factor affecting the exchange rate. However, quite a lot of variability in the exchange time remained unaccounted. The actual measured exchange differed up to 110% from the predicted by the power relationship with the delay time. However, as long as the experimental conditions remained unchanged, there was very little variation in the response (see below).

### Interface behavior at the sub-optimal zone

**Fig 5:**
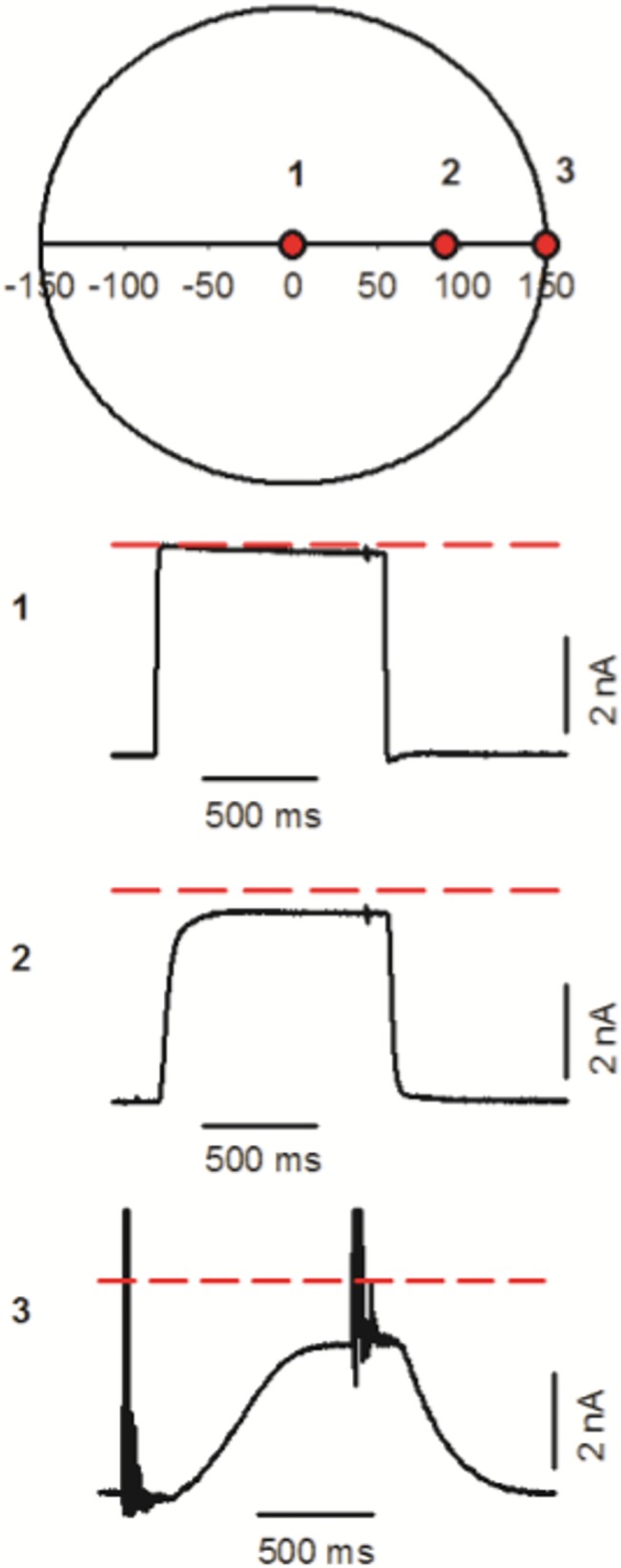
The effect of the off-center distance. The upper diagram is a schematic representation of a cross section of the stream. Three different recording positions are shown (red circle 1 is limit zone, red circle 2 is optimal position and red circle 3 is a suboptimal position). 1; 2; and 3 are the records of each position and dashed red line indicates the maximum response at all positions.

In this section, we analyze how the interface behaves outside of the optimum point. To minimize the delay time we worked with a T-junction placed at 1 mm of the application tip and we used 0.4 bar of N2. At each recording position, a pulse of 1 sec was delivered to measure the exchange dynamics. Fig 5 shows a schematic representation of the transversal section of the stream with three recording points in the horizontal axis. At the optimal point (point 1) the delay time was minimal (19 ms). It increased 6 times (118 ms) when the pipette was displaced 100 μm (point 2), and 15 times (288 ms) when the pipette was moved close to the border of the solution stream (point 3). The exchange time, on the other hand, was 0.49 ms at point 1, 180 times longer at point 2 (87 ms) and 1240 times longer at point 3 (601 ms). The amplitude of the recordings indicated the extent of the exchange fulfilled. This was nominally 100% at point 1, 90% at point 2 and only 68% at point 3. Small oscillations in the measured current appeared when the valve was switched from test to control solution at points 1 and 2. These oscillations were much bigger at point 3, and appear also at the initial valve switch.

**Fig 6:**
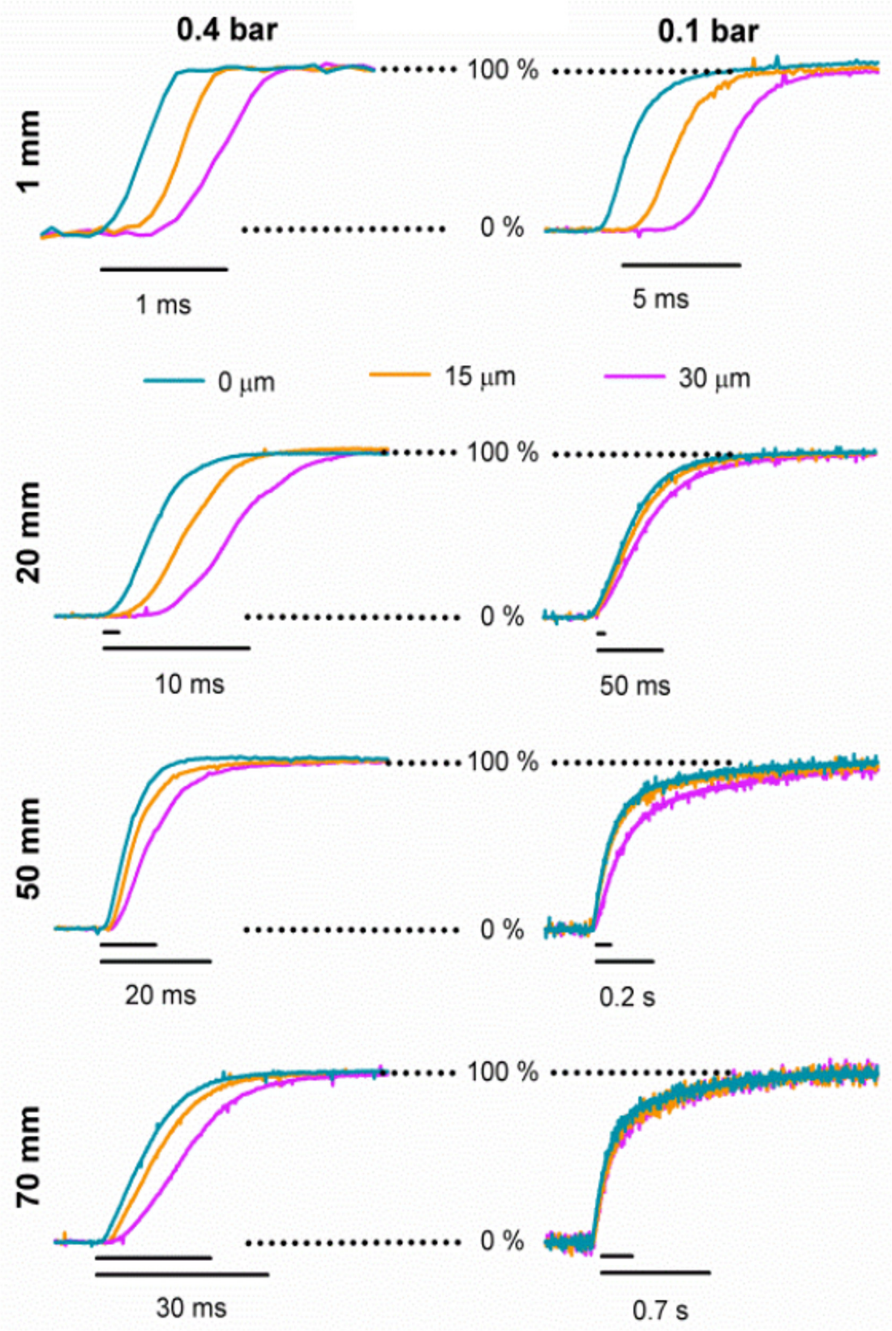
Time course of the solution exchange slowed down away from the optimal position. Exchanges were measured at 0, 15 and 30 μm away from the optimal position for 0.1 or 0.4 bar and for distances of 1, 20, 51, or 70 mm. The small line above each scale bar indicates the scale bar of the previous distance.

Next, we analyzed the behavior in the vicinity of the optimal point. Thus, we placed the recording pipette at 0, 15 or 30 μm from the optimal point and recorded pulses as above (Fig 6). At all distances and pressures tested, the delay and the exchange times increased as the recording pipette moved away from the optimal point (Fig 6).

**Fig 7:**
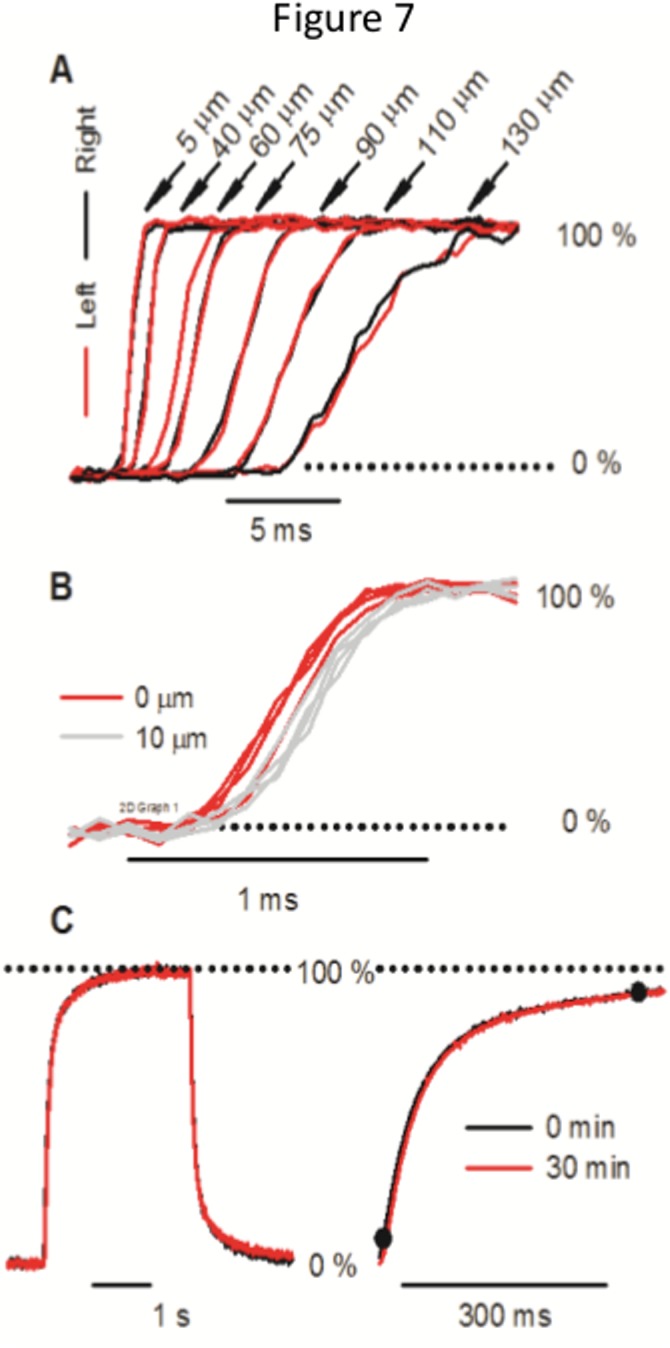
Symmetry and variability of the forward exchange. (A) Symmetric change in the exchange at increasing off-center distances. Left and right from the optimal point were plotted in red or black respectively at 1 mm of travelled distance and 0.4 bar. (B) Trace to trace variability at the optimal point (black) and 10 micrometers off-center (violet). (C) Recording stability. At the optimal point two traces that differ 30 minutes were compared. Traces were generated using T-junction at 51 mm and 0.1 bar.

Finally, we analyzed symmetry and variability for 1 mm and 0.4 bar the stability of the recordings for 51 mm and 0.1 bar. A clear symmetry from the central point of stream was evident (Fig 7A) at least up to 75% of the stream width (130 μm) at 1 mm and 0.4 bar. The variability between successive traces at the same position was in the order of the changes measured after moving the pipette a distance of 10 μm. No differences were found between 0 and 10 μm neither on the exchange time (464.5 ± 52.1 μs vs. 548.5 ± 71.6 μs NS) nor on the delay time (17.44 ± 0.07 ms vs. 17.48 ± 0.05 ms p = 0.19 with t-test; Fig 7B). Thus, a positioning error of 10 μm from the central point would generate an error in the order of 0.1 ms in the time of the solution exchange. We found almost no difference on the temporal profile of exchanges separated by 30 min (Fig 7C). The delay time increased by 6 ms (1.1% of the 554 ms delay time). The exchange time increased by 12 ms (1.6% of the initial 734 ms exchange time). This slight increase in the delay and exchange time could be explained by a small reduction in the propelled pressure.

**Fig 8:**
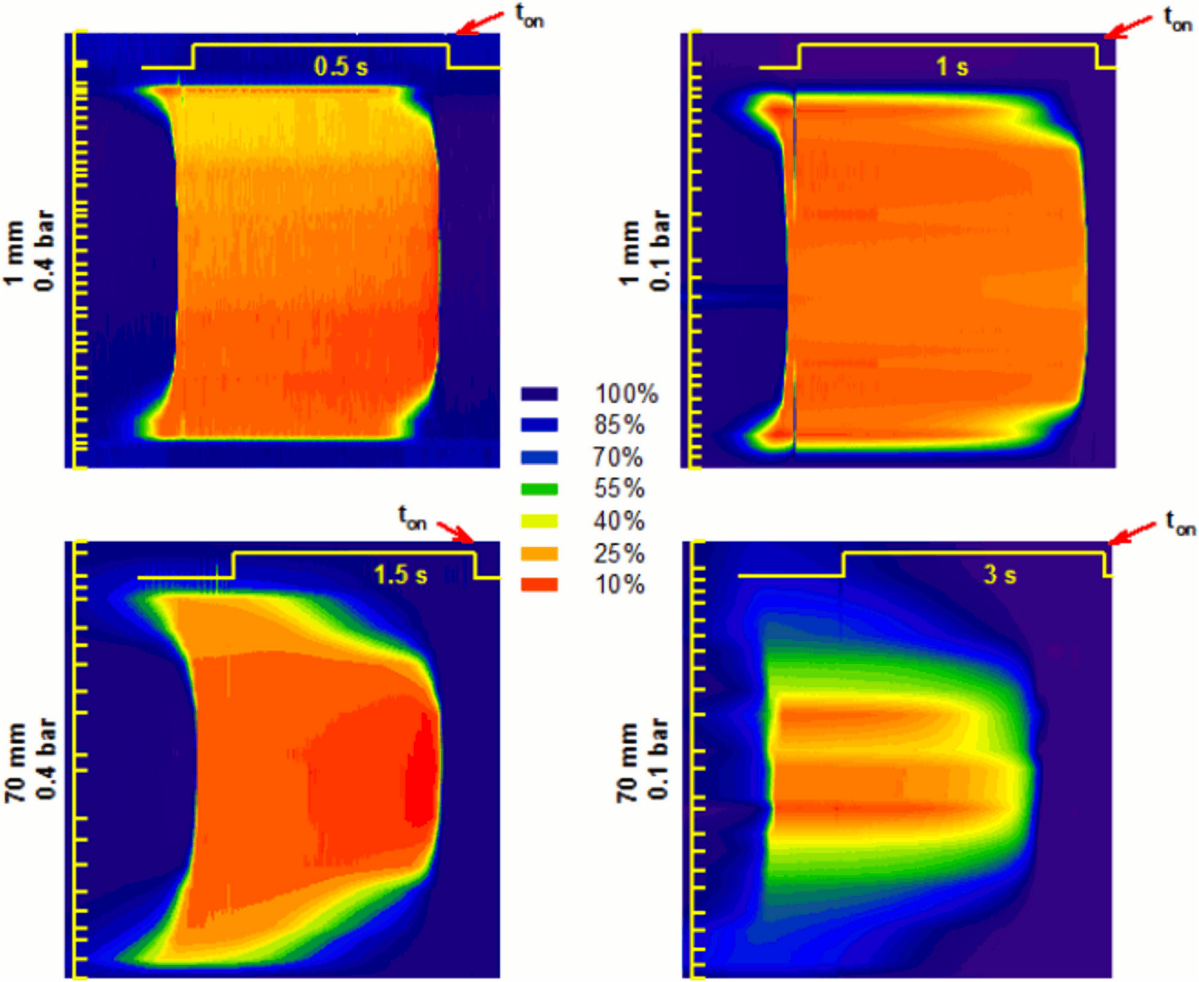
Sagittal section of the time course of the solution exchange. The vertical yellow bar represents 350 μm; ticks indicate the position of each recorded trace used to interpolate the data. The yellow horizontal traces indicate the command voltage applied to the valve, (notice that the time increases from right to left. In this way the plot gives the right impression that the center goes faster than the borders). Red arrows indicate the ton. The diffusive nature of the profile is clearly increasing with distance and lowering pressure.

Fig 8 shows the space profile of the solution exchange dynamics at different conditions. This Fig represents the evolution of the solution exchange recorded after a pulse of test solution was applied (Fig 8: yellow pulse at the top of each graph) at different recording positions (Fig 8: yellow tics) over a line perpendicular to the stream. Increasing travelling distance and decreasing pressure resulted on a greater effect of the relative position of the pipette on the exchange dynamics. The exchange was slower at peripheral zone and much faster at the central zone. This effect was more evident at the lower pressure and longer travelled distance.

## Discussion

The experimental study of drug interactions on ion channels preparations depends on a proper way to apply an arbitrary number of compounds. In pharmacological studies a relevant issue consists in screening a large number of compounds, while in biophysics, a careful characterization of a single compound is the basis for understanding its action mechanism [31, 32]. The gold standard for the study of ion channels pharmacology and a fundamental tool in ion channels biophysics is the patch clamp. Patch clamp has a stunning time resolution: up to few milliseconds in whole cell [14, 19, 20] or tens of microseconds in the outside out configuration [8, 9]. In order to take advantage of this amazing resolution it is necessary to gain control of the time course of the studied compound with the same time accuracy. Up to now, this time scale was only possible by using downstream exchangers where a stationary interface between control and test solutions has been build.

In present paper we analyzed the dynamics of solution exchange in a T-junction system. We found that (1) it is possible to manufacture inexpensive T-junctions with standard silicone and polyethylene tubes. (2) Stable solution exchanges in a wide variety of exchange rates from 0.5 ms to 600 ms could be generated by using those T-junctions. (3) Three factors, pressure, travelled distance and off-center distance govern the timing of solution switching.

We suggest that a serial solution exchanger could be built of T-junctions. The advantages of the proposed exchanger would be (1) the possibility of easily studying the dose-response curve of the compound of interest in a faster and more efficient way and (2) the possibility of studying the interactions among different compounds acting on the same ion channel while (3) being able to switch dynamically from one compound to another according to the observed interactions.

### Practical Limits for Solution Exchange Upstream the Exit Port

In this paper we investigated the practical limits of upstream exchangers for controlling the time course of compound applications. Specifically, we set up simple T-junctions that allow alternating solutions of different composition each controlled by an independent solenoid valve. We presented a simple way to manufacture T-junctions over a single silicone tube and we characterized their dynamics.

A linear relationship between the time delay and the travelled distance was found for each tested pressure, showing that the delay was determined by the fluid velocity, which, as expected, increased with pressure. A log-log plot indicated that the increase in the exchange time with increasing distance or decreasing velocity seemed to be explained by the increase in the delay. In other words, doubling the delay resulted in approximately quadrupling the exchange time, either by doubling the distance or by halving the velocity. On the other hand, a sizeable variability remains unexplained by this relationship. We hypothesize that this variability might arise because of a complex, but stable and not chaotic, mixture pattern due to the presence of the manufactured T-junctions. This quadratic relationship allows estimating the combination of fluid velocities and travelled distances compatible with a desired exchange time. However, the precise exchange time has to be experimentally determined in each case. Those exchange times were quite stable for each particular configuration. This stability is consistent with the laminar flow regime that the low Reynolds number of the system indicates.

We also found that the solution switching timing was strongly dependent on the position of the recording pipette relative to the center of the stream. The delay and exchange times increased as the pipette moved away from the center of the stream. This spatial heterogeneity put a limit on the size of patch, cell or any biological sample to be exposed to the exchange of solution. In the case of the 0.3 mm ID tubing analyzed here it appears to be around 20 micrometers at the center of the stream. Using a larger biological sample or positioning it off-center would result in the stimulus to be spatially heterogeneous.

Near the stream borders the delay and exchange times increased dramatically. According to fluid mechanics, the fluid velocity tends to zero as we get close to the tube walls and it increases quadratically as we depart from them. This fact would explain the increased delay at the stream border. Furthermore, mass transport between the slow fluid at the border and the fast fluid at the center seems to be responsible for both the term containing the exponential of the square root of time, which is needed to fit the time course of the solution exchange as well as the quadratic relationship between exchange time and delay. This relationship contrasts with the predictions of a purely diffusive process, where the increase in the exchange time would be with the square root of the delay. In this scenario, it seems unlikely that differences in the diffusion coefficient of different compounds would result in big differences in the temporal profile of their exchange. Further studies would be necessary to clarify this issue.

It is likely that the weight of the term containing the exponential of the square root of time would be bigger on compounds that tend to be adsorbed onto the tubing walls. That would mean slower exchange times for those compounds. This would be a likely scenario in the context of drug discovery, where most of the new compounds are highly hydrophobic bulk molecules which might stick to or block narrow silicone tubing. This problem might be solved by using commercially available polytetrafluoroethylene (PTFE) or polyetheretherketone (PEEK) tees and micro-fittings that are commonly used in HPLC applications. Regular cleaning of the tubing is mandatory to avoid an increased adsorption of the tubing walls. Several factors would limit the stability of the exchange system: 1) The mechanical stability of relative position of the application pipette relative to the patch pipette. However, provided that the center of the stream is accurately located, the low drift granted by commercially available patch pipette positioners (1 micron per hour) guarantee to remain inside the safety zone (i.e, 10 micrometer around the center) during the course of the experiment; 2) The stability of the propelling pressure. Because of the quadratic dependence, we would need 0.5% of pressure stability to achieve 1% of exchange time stability; 3) The presence of bubbles inside the solution exchanger has to be prevented. Degasing of the solutions and purging the system by applying solutions at high pressure is fundamental. The performance of the T-junctions might be affected by several factors as well: 1) Changes in the adsorption of the tubing due to accumulation of hydrophobic compounds, algae or bacteria; 2) Changes in the mechanical properties of the tubing due to degradation or swelling of the tubing walls; 3) Malfunction of the controlling valves; 4) Leakage in the applied pressure or in the exchange system. Periodic cleaning and replacement of the tubing as well as monitoring valves performance and system pressure are therefore mandatory.

### Performance and stability of T-junction systems

When the traveled distance is very short (1 mm), the measured exchange time of the T-junction was 0.5 ms. Although this value is far from their state of the art (0.03 ms) [11], it is in the temporal range that allows to separate binding from gating in receptors with slow activation such as GABAc and NMDA [33, 34]. This is the range of commercially available ultra-fast downstream exchanger systems that includes the valve driven Y exchanger [35, 36] which is structurally not very different from a 1 mm T-junction.

The exchange times for T-junctions at longer traveled distances are at least ten times slower (> 5 ms) and it is similar to the performance of the commercially available Multi-barrel perfusion pencil. A 0.25 mm Perfusion Pencil flowing 1.6 ml/min (a flow rate used for whole cell recordings, [37, 38]) will deliver solution at the same velocity that our tested T-junction at the highest tested pressure. At this velocity a 38 mm application tip would take 67 ms to be traveled, and Fig 4C would predict an Exchange Time of 2–16 ms, something that is compatible with manufacturer claims (10 ms) and published experimental results [30]. On the basis of present work we strongly recommend Perfusion Pencil users to carefully position the sample in the center of the stream. Furthermore, its exchange rate would be much faster if shorter application tips are used, at least in theory. Actually, given the shape of the solution mixer, it is possible that the mixing might not be completed at those shorter distances, spatially and temporally heterogeneous patterns might arise, that might be different for each mixed pair.

### A T-junction based Serial Solution Exchanger

Planar patch clamp technology [39] allowed for the first generation of automatic patch clamp devices [22]. Those devices allow working in parallel with hundreds of samples at the same time [40]. Another possibility for increasing the data acquisition rate consists in using a serial solution exchanger to try hundreds of compounds in succession over a single sample [14]. In this way it would be possible to perform studies of the kinetics of the reciprocal interactions between drugs, which requires a large number of experiments. For example multi-laminar flow streams devices allow to test 32 compounds in 40 s on a single patch [26] while perfusion pencil can serially apply 4–16 compounds with a 10–50 ms of maximal exchange rate [30]. On the other hand, Ion Flux devices use up to 8 upstream microfluidic exchangers colliding on each one of up to 20 planar patches. This system needs 100 ms to change of compound and it can only test for 8 compounds on each plate.

A Serial Solution Exchanger based on a linear array of 100 T-junctions would be theoretically possible to accommodate in 10 cm; we have successfully placed two T-junctions 1 mm apart. Using a flow rate of 0.5 m/s, the expected delay of such device would be less than 200 ms which would complete the exchange in less than 14–120 ms (Fig 4*C*). Assuming a data cycle of 3 s and a patch survival of 20 minutes, testing 100 compounds per patch would be feasible.

Besides primary screening, serial solution exchangers would help to study the kinetics of the interaction between different drugs on the same target. If implemented, this proposed method would allow such studies since it would be possible to dynamically change the drug protocol according to the obtained results, something that Multibarrel Perfusion Pencil systems would be able to do for up to 16 compounds. Multilaminar stream devices can manage up to 32 compounds but they lack the ability to dynamically change the order of drug application.

It is important to note that some drugs would induce irreversible changes in the channel response. Drugs that completely abolish the response would effectively halt the data acquisition for that particular patch preparation. Therefore, experimental protocols that include such drugs should be avoided. But reversible interactions could be effectively studied with a serial solution exchanger. The analysis of the generated data would be less straightforward as it should explicitly consider the possibility of the drugs to bind the target and changing the responsiveness of the ion channel to a subsequent drug.

In summary, the proposed serial solution exchanger could be built of T-junctions and it would present several advantages (1) a faster and more efficient study of the dose-response curve of the compound of interest (2) the ability to study the interactions among different compounds acting on the same ion channel (3) the faculty to dynamically change the order in which the tested compounds are tested according to the observed interactions. The ability to do this kind of experiments would be of great value for the discovery of drugs that interact with the natural agonists of ion channels with the purpose of fine tuning their physiology.
